# Longitudinal comparison of direct medical cost, radiological and health-related quality of life treatment outcomes between traditional growing rods and magnetically controlled growing rods from preoperative to maturity

**DOI:** 10.1186/s12891-022-05750-7

**Published:** 2022-08-18

**Authors:** Prudence Wing Hang Cheung, Carlos King Ho Wong, Jewel T. Sadiang-abay, Sin Ting Lau, Jason Pui Yin Cheung

**Affiliations:** 1 Department of Orthopaedics and Traumatology, The University of Hong Kong, 5/F, Professorial Block, Queen Mary Hospital, Pokfulam, Hong Kong SAR China; 2 Department of Family Medicine and Primary Care, The University of Hong Kong, Ap Lei Chau, Hong Kong SAR China

**Keywords:** MCGR, TGR, Early onset scoliosis, Direct cost, HRQoL, Treatment outcomes

## Abstract

**Background:**

Magnetically controlled growing rods (MCGR) have replaced traditional growing rods (TGR) in the past decade, however, a comparison of their direct costs and treatment outcomes based on real longitudinal data is lacking. This study aims to compare the direct cost and treatment outcomes between TGR and MCGR, whilst incorporating complications, reoperations and changes in health-related quality of life (HRQoL) throughout the entire treatment course.

**Methods:**

Patients with early onset scoliosis (EOS) who underwent initial growing rod surgery between 2003 and 2016 at a tertiary scoliosis clinic were studied with longitudinal data. Accumulated direct medical costs were calculated based on the unit cost of surgeries of each TGR and MCGR, costs incurred for any rod exchange or remedial surgery for post-operative complication. Treatment outcomes were evaluated via: Patient’s HRQoL using SRS-22r questionnaire, and radiological parameters (including major curve correction, spine length gains, spinal balance) throughout the treatment until maturity.

**Results:**

A total of 27 EOS patients (16 MCGR, 11 TGR) were studied. Total direct cost of index surgery for MCGR was HKD$223,108 versus lower cost of HKD$135,184 for TGR (*p* < 0.001). At 2–3 years post-index surgery, accumulative total direct medical cost of MCGR and TGR became most comparable (TGR:MCGR ratio = 1.010) and had reached neutrality between the two groups since. Radiological parameters had no intergroup differences at maturity. For HRQoL, TGR group had shown the trend of less pain (domain score mean difference: 0.53, *p* = 0.024) post-index surgery and better self-appearance (domain score mean difference: 1.08, *p* = 0.017) before fusion. Higher satisfaction with treatment (domain score mean difference: 0.76, *p* = 0.029) was demonstrated by TGR patients at fusion/maturity. MCGR had negative (*r*_*s*_ = -0.693) versus TGR’s positive (*r*_*s*_ = 0.989) correlations (*p* < 0.05) of cost and SRS-22r total scores at 2–3 years post-index surgery.

**Conclusions:**

From index surgery to maturity, TGR demonstrated better satisfaction with treatment by patients and comparable overall HRQoL with MCGR *during* the treatment course, as MCGR did not show apparent benefit despite less surgeries and cost neutrality between the two groups at 2–3 years post-index surgery.

## Introduction

Traditional growing rods (TGR) have been the mainstay treatment for patients with early onset scoliosis (EOS) since they were introduced in the 1980s. However, these patients require multiple surgeries under general anaesthesia until each patient reaches skeletal maturity, and often result with various anaesthetic and surgical complications [1]. In the past decade, magnetically controlled growing rods (MCGR) have replaced TGR with equal clinical results, and less radiation and surgical risks during their course of treatment [2–11]. However, with reports of unique complications such as distraction failure and metallosis, the actual benefits of long-term MCGR use have been questioned [12–18].

Healthcare economics is an important factor to be considered when designing management programs with treatment options. A balance needs to be maintained between the cost of the procedure and the potential treatment outcomes. Despite a certain treatment restoring good health-related quality of life (HRQoL) outcomes, it may not be an effective option for all patients or may quickly exhaust the resources available in the medical system.

Current literature of prospective direct cost comparison between TGR and MCGR is scarce, only establishing economic models with projected estimation of costs through time [19, 20]. Real long-term data for comparing actual outcomes of growing rod graduates, considering of complication rates, and its remedial treatment and costs incurred is lacking. Now a decade since its inception, it is timely to study the cost and its corresponding treatment outcomes of MCGR from initial implantation till graduation. This study aims to perform a prospective direct cost comparison between TGR and MCGR whilst incorporating complications, reoperations and changes in HRQoL.

## Patients and methods

### Study design

This was a prospective study of patients with EOS who underwent initial growing rod surgery between 2003 to 2016. The selection criteria for TGR and MCGR was the same: any patients who were aged above 5 years but below 10 years with either a scoliosis of major coronal curve > 50° or documented > 10° spinal deformity progression in one year; or patients aged ≤ 5 years with scoliotic major curve > 30° degrees and a documented curve progression of > 10° in one year. Patients who required regular magnetic resonance imaging (MRI) postoperatively were not treated with MCGRs. All patients were followed until graduation and final fusion surgery. Longitudinal data collection was performed from pre-operative and at index surgery, at each visit including each subsequent distraction, rod exchange or remedial surgery for complications during follow-up. Patients were divided into TGR and MCGR groups. Cost of each TGR and MCGR were based on unit cost of surgeries and cost incurred for any complication postoperatively. Surgical outcomes were defined via patient’s HRQoL as well as radiographic parameters to be elaborated on later. Time-points for intergroup comparison of treatment outcomes were: preoperative, immediate postoperative, subsequent follow-up visits and at final fusion/maturity. Ethics approval was obtained from the local ethics committee and with parental consent gained.

### Cost analysis

All unit cost (in HKD, and equivalent USD values) for each service component associated with the use of growing rod were retrieved from the Department of Orthopaedics and Traumatology, the University of Hong Kong at its affiliated hospitals: The Duchess of Kent Children’s Hospital and Queen Mary Hospital. Unit costs for surgery included the cost of growing rods (TGR and MCGR), cross-links and hooks/screws, operating theatre (including staff costs – anaesthetist, orthopaedic surgeons, theatre nurse; drugs – antibiotics and anaesthesia, dressings and consumables), intraoperative spinal cord monitoring, length of hospitalization (cost of hospital stay in intensive care unit and general ward) and the use of imaging (Table 1). Subsequent postoperative outpatient visits and consultation fees for MCGR distraction, and treatment required for dealing with complications were also calculated. Costs associated with radiology, hospitalizations, outpatient, and physiotherapy visits were based on the price list in the government gazette [21], which itemized the charges to non-Hong Kong residents for use of health services in the Hospital Authority.

**Table 1:** Unit cost (HKD and equivalent USD values) for each service component associated with the use of growing rods in early onset scoliosis

	Unit cost $HKD	Equivalent $USD	Reference
**Growing rod**			
Single MCGR	58,500	7455.3	Nuvasive®
Dual MCGRs	117,000	14,910.6	Nuvasive®
Traditional growing rod	27,528	3508.3	Total cost for construct Medtronic® CD Horizon® Legacy^TM^ system
Legacy	27,528	3508.3	Medtronic® CD Horizon® Legacy^TM^ system
Cross link	1500	191.2	Medtronic® CD Horizon® Legacy^TM^ system
4 Hooks	5580	711.1	Medtronic® CD Horizon® Legacy^TM^ system
4 Screw	6732	857.9	Medtronic® CD Horizon® Legacy^TM^ system
4 Set screws	2896	369.1	Medtronic® CD Horizon® Legacy^TM^ system
Rod	3090	393.8	Medtronic® CD Horizon® Legacy^TM^ system
Rod cross connector	6786	864.8	Medtronic® CD Horizon® Legacy^TM^ system
**Surgical treatment**			
Spinal implants used	35,000	4460.3	Department of O&T, HKU
Spinal cord monitoring	629	80.2	Department of O&T, HKU
Intensive care unit, per night	23,000	2931	Government Gazette
General ward, per night	4680	596.4	Government Gazette
**Operating theatre**			
Salary of staff	37,989.75	4841.2	Department of O&T, HKU
Drugs	3792.62	483.3	Department of O&T, HKU
Consumables	2733.99	348.4	Department of O&T, HKU
Dressing	325.16	41.4	Department of O&T, HKU
**Radiology**			
Imaging examination	566	72.1	Department of O&T, HKU
**Outpatient visits**			
Salary of staff	1,859.34	237	Department of O&T, HKU
Consultation	1110	141.5	Government Gazette
**Routine follow-up visit**			
Outpatient visits			
Salary of staff	464.83	59.2	Department of O&T, HKU
Consultation	1110	141.5	Government Gazette
Physiotherapy visit	1050	133.8	Department of O&T, HKU
**P&O visit**			
Corset (first time)	3170	404	Department of O&T, HKU
Adjustment (first time)	700	89.2	Department of O&T, HKU
**Complications from Surgical treatment**			
Infection			
Superficial	221.2	28.2	Department of O&T, HKU
Deep	96,640.40	12,315.6	Department of O&T, HKU
Implant pullout	84,967.50	10,828.1	Department of O&T, HKU

*P&O* Prosthetics and orthotics, *MCGR* Magnetically controlled growing rod, *TGR* Traditional growing rod,*HKD* Hong Kong Dollar, *O&T* Orthopaedics and traumatology, *HKU* The University of Hong Kong

### Clinical and radiological parameters

Clinical parameters and patient demographics including age at index surgery, gender, diagnosis and nature of scoliosis, ambulatory status and comorbidities were recorded. Patient-perceived HRQoL was assessed using the refined Scoliosis Research Society 22-item (SRS-22r) questionnaire [22, 23].

Radiological parameters included measurements related to the effectiveness of growing rod surgery for spinal deformities. Deformity correction was assessed by the coronal Cobb angle of the major curve. Any deterioration in Cobb angle exceeding 5 degrees (ͦ) throughout subsequent follow-ups was considered unfavourable. Overall balance was studied by the coronal balance as measured by C7-CSVL and trunk shift, and by sagittal balance as measured using the sagittal vertical axis. For spine length gains, T1-12 and T1-S1 spine lengths were measured on posteroanterior spine radiographs between the perpendicular levels at the midpoint of upper endplate of T1 and at the midpoint of lower endplate of T12 (or S1). Global kyphosis and lumbar lordosis were also examined for intergroup comparison. Any coronal balance with absolute values of C7-CSVL and trunk shift < 20 mm, and sagittal balance between + 50 mm to -50 mm were considered as good surgical outcomes [24, 25].

### Statistical analysis

Descriptive statistics were presented in mean values and standard deviations, counts and percentages, with 95% confidence intervals (CI) where appropriate. Comparisons of patient’s demographics, distribution of nature of scoliosis diagnosed, and the occurrence of comorbidities between MCGR and TGR groups were performed using independent samples *t*-test and chi-square test/Fisher’s exact test. Normality tests were performed via Shapiro–Wilk tests. Accumulative total direct medical cost was compared between the two groups by independent samples *t*-tests at index surgery, index surgery year, and each subsequent year up to final fusion. HRQoL was analysed for intergroup differences through comparing the domain and total scores of SRS-22r at multiple time-points. The changes of scores between time-points were also compared to determine whether there were significantly more changes of quality of life in one study group than the other. Radiological parameters were analysed with the same approach for any intergroup difference at pre- and post-operative of index surgery and at final fusion, and the magnitude of changes between time-points were compared. In addition, the count of good surgical outcomes based on coronal and sagittal balance were investigated. For the assessment of cost and treatment outcomes, the relationship between accumulative total direct medical cost and HRQoL at the corresponding time points was tested using the Spearman rank-order correlation test with correlation coefficient (*r*_s_) indicating the strength of relationship [26]. All patients were followed post-final fusion and were assessed if any additional surgeries were needed until the last follow-up at the clinic. Statistical analyses were performed using STATA version 16.0 (StataCorp LP. College Station, Texas, USA) and SPSS 26.0 (IBM SPSS Inc., Chicago IL, USA). Post-hoc power analyses were conducted using G*Power (version 3.1.9.4; Heinrich-Heine-Universität Düsseldorf, Düsseldorf, Germany). A *p*-value of less than 0.05 was considered statistically significant.

## Results

**Fig. 1:**
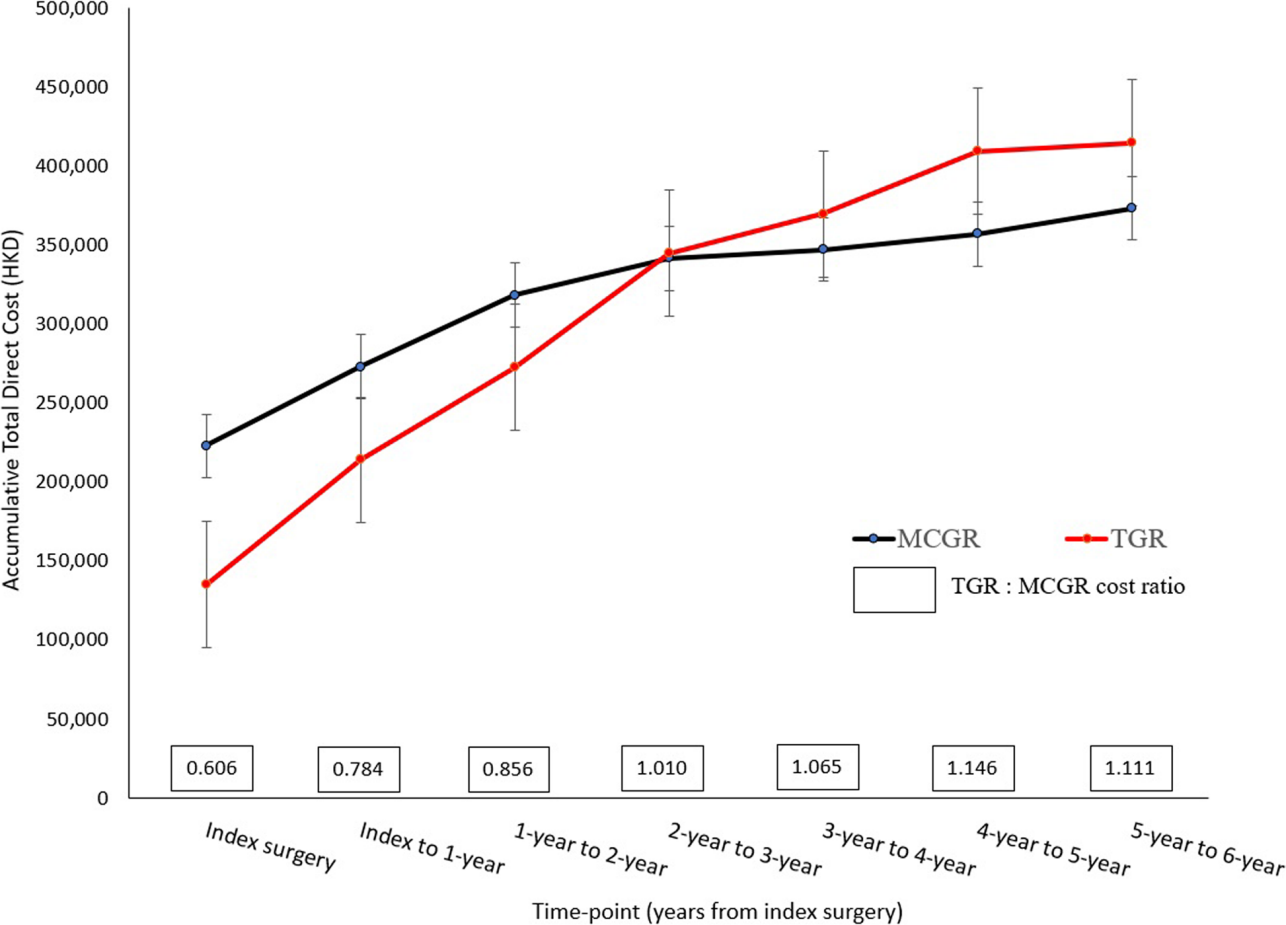
Accumulative total direct costs of MCGR and TGR groups from index surgery through time

A total of 27 EOS patients (82% females) were studied, with a mean age of 9.6 ± 3.5 years at index surgery (Table 2). 16 patients had received MCGR and 11 patients had TGR surgery. The direct cost of index surgery revealed a total of HKD$223,108 (USD$28,427) for MCGR versus a significantly lower cost of HKD$135,184 (USD$17,225) for TGR (*p* < 0.001) (Table 3). Throughout the index surgery year, the accumulative total direct cost was lower for TGR group (*p* = 0.025), and this trend continued at the first to second year post-index surgery, with *p*-value marginally short of statistical significance (HKD$272,827 (USD$34,762) of TGR group versus HKD$318,551 (USD$40,588) of MCGR group, *p* = 0.065). At 2 to 3 years post-index surgery, the cost ratio of TGR:MCGR was 1.010. From 2–3 years post-index surgery onwards, TGR group had higher total direct cost and remained comparable with the cost of MCGR group (Fig. 1). Patients were followed for 4.5 ± 2.5 years after final fusion, with none of them requiring any additional surgeries post-fusion (Table 2).

**Table 2:** Patient profile of the study cohort

	Whole cohort (*N* = 27)	MCGR (*N* = 16)	TGR (*N* = 11)	*p* value
Demographic Variables				
Age at index surgery years, mean (SD)	9.6 (3.5)	10.6 (3.4)	8.1 (3.2)	0.064
Female sex	22 (81.5)	14 (87.5)	8 (72.7)	0.332
Ambulatory	25 (92.6)	15 (93.8)	10 (90.9)	0.234
Diagnosis				
Syndromic Scoliosis	2 (7.4)	2 (12.5)	0 (0.0)	0.499
Neurofibromatosis	3 (11.1)	2 (12.5)	1 (9.1)	1.000
Neuromuscular Scoliosis	3 (11.1)	1 (6.25)	2 (18.2)	0.549
Idiopathic Scoliosis	13 (48.1)	11 (68.75)	2 (18.2)	0.010*
Congenital Scoliosis	6 (22.2)	0 (0.0)	6 (54.5)	0.002*
Comorbidities				
Developmental Delay	3 (11.1)	3 (18.8)	0 (0.0)	0.128
Neurofibromatosis	4 (14.8)	2 (12.5)	2 (18.2)	0.683
Hearing Impairment	3 (11.1)	1 (6.3)	2 (18.2)	0.332
Additional surgeries after implant insertion				
Yes: No	7:27	3:16	4:11	0.305
Number of additional surgeries	11	5	6	0.807
Additional implants required	4	2	2	1.000
Complications				
Wound infection requiring debridement	4	0	4	0.085
Infection with implant loosening	1	1	0	
Screws/implant loosening	1	1	0	
Failed rod distraction	1	1	0	
Proximal junctional kyphosis	7	5	2	
Final Fusion				
Age years, mean (SD)	15.4 (2.6)	16.0 (2.6)	14.5 (2.4)	0.127
Duration of follow-up since fusion years, mean (SD)	4.5 (2.5)	3.6 (2.2)	5.7 (2.4)	0.053
Age at last follow-up years, mean (SD)	19.0 (3.8)	18.9 (3.4)	19.2 (4.4)	0.806
Additional surgeries post-fusion	0	0	0	-

^*^ Statistical significance at *p* < 0.05

**Table 3:** Mean and standard deviation of direct medical costs in each patient of MCGR and TGR

		Whole cohort (*N* = 27)	MCGR (*N* = 16)	TGR (*N* = 11)	TGR vs MCGR			
Time-points		Accumulative total direct medical cost						Power^
		Mean (SD)	Mean (SD)	Mean (SD)	Cost ratio	95% C.I	*p* value	
Index surgery	HKD	187,287 (64,963)	223,108 (21,204)	135,184 (72,519)	0.606	(0.464, 0.791)	< 0.001*	0.99
	USD	23,864 (8,277)	28,427 (2,702)	17,225 (9,240)				
Index to 1-year	HKD	249,174 (68,515)	273,197 (45,199)	214,231 (82,911)	0.784	(0.634, 0.970)	0.025*	0.63
	USD	31,749 (8,730)	34,810 (5,759)	27,296 (10,564)				
1-year to 2-year	HKD	299,923 (65,536)	318,551 (56,000)	272,827 (71,415)	0.856	(0.726, 1.010)	0.065	
	USD	38,214 (8,350)	40,588 (7,135)	34,762 (9,099)				
2-year to 3-year	HKD	343,091 (73,647)	341,722 (59,220)	345,084 (93,991)	1.010	(0.854, 1.194)	0.909	
	USD	43,714 (9,383)	43,539 (7,545)	43,970 (11,976)				
3-year to 4-year	HKD	356,534 (80,628)	347,378 (58,363)	369,852 (107,068)	1.065	(0.896, 1.265)	0.476	
	USD	45,428 (10,273)	44,262 (7,436)	47,123 (13,642)				
4-year to 5-year	HKD	378,469 (109,515)	357,153 (66,959)	409,474 (150,578)	1.146	(0.929, 1.415)	0.203	
	USD	48,222 (13,954)	45,506 (8,531)	52,173 (19,186)				
5-year to 6-year	HKD	390,332 (106,215)	373,448 (55,765)	414,892 (153,454)	1.111	(0.909, 1.358)	0.304	
	USD	49,734 (13,533)	47,582 (7,105)	52,862 (19,552)				

Cost ratio = TGR: MCGR costs

^*^ Statistical significance at *p* < 0.05

^ Post-hoc power analyses with α = 0.05, two-tailed, and effect size calculated for the variable at the specific time-point

For surgical outcomes in terms of HRQoL, the SRS-22r total and domain scores were comparable between the TGR and MCGR groups though mean values were generally higher for the TGR group at almost all time points (Table 4). Mean total score was higher for TGR at all time-points, with 4.66 ± 0.12 of TGR versus 4.22 ± 0.32 of MCGR at the follow-up prior to final fusion/maturity being just short of statistical significance (*p* = 0.053). TGR had higher Function domain score (4.69 ± 0.34 versus 4.37 ± 0.31, *p* = 0.051) and Pain domain score (4.83 ± 0.37 versus 4.23 ± 0.68, *p* = 0.049) immediately post-surgery than the MCGR group. The changes of HRQoL immediately after surgery was found comparable between the two groups. The TGR group had more worsening of Pain domain score (*p* = 0.024) than MCGR group at first follow-up as compared to immediately post-index surgery. However, the TGR group had greater improvement in Appearance domain score of 1.08 than the MCGR group at follow-up before final fusion/maturity (*p* = 0.017) but no difference at final fusion/maturity. But both these differences of change of the Pain and Appearance domain scores lacked the power due to the sample size. The TGR group had greater improvement in Satisfaction with Treatment domain score than MCGR group at final fusion/maturity when compared to immediately post-index surgery by an intergroup difference of 0.76 (*p* = 0.029, power: 0.97).

**Table 4:** Mean and standard deviation of SRS domain and total scores for patients in MCGR and TGR groups

	Whole cohort (*N* = 27)	MCGR (*N* = 16)	TGR (*N* = 11)	*p* value	Difference in changes (postop – preop) of TGR vs MCGR	*p* value	Difference in changes (FU—post-surgery)_TGR_ minus (FU—post-surgery)_MCGR_	*p* value	Power^
Time-points	SRS-22r domain scores								
	Function								
Pre-surgery	4.53 (0.37)	4.50 (0.48)	4.60 (0.00)	0.793					
Post-surgery	4.48 (0.35)	4.37 (0.31)	4.69 (0.34)	0.051	0.25	0.578			
FU1	4.16 (1.18)	4.30 (0.37)	3.93 (1.95)	0.564			-0.73	0.287	
FU2	4.31 (0.39)	4.26 (0.44)	4.45 (0.19)	0.432			-0.27	0.126	
FU3	4.43 (0.42)	4.30 (0.44)	4.70 (0.20)	0.120			0.03	0.907	
FU4	4.38 (0.37)	4.28 (0.38)	4.67 (0.12)	0.126			0.08	0.577	
Final fusion/ maturity	4.38 (0.49)	4.23 (0.51)	4.73 (0.23)	0.147			0.20	0.502	
	Pain								
Pre-surgery	4.73 (0.35)	4.60 (0.37)	5.00 (0.00)	0.218					
Post-surgery	4.45 (0.65)	4.23 (0.68)	4.83 (0.37)	0.049*	0.30	0.740			
FU1	4.63 (0.36)	4.58 (0.42)	4.71 (0.24)	0.494			-0.53	0.024*	0.15
FU2	4.51 (0.73)	4.42 (0.85)	4.75 (0.19)	0.465			-0.53	0.242	
FU3	4.67 (0.37)	4.63 (0.41)	4.75 (0.30)	0.601			-0.55	0.127	
FU4	4.73 (0.34)	4.68 (0.37)	4.87 (0.23)	0.431			-0.48	0.233	
Final fusion/ maturity	4.66 (0.75)	4.51 (0.87)	5.00 (0.00)	0.378			-0.20	0.765	
	Appearance								
Pre-surgery	3.77 (0.70)	4.05 (0.25)	3.20 (1.13)	0.181					
Post-surgery	3.89 (0.71)	3.93 (0.65)	3.83 (0.86)	0.767	0.40	0.742			
FU1	3.85 (0.48)	3.84 (0.42)	3.88 (0.61)	0.883			0.25	0.438	
FU2	3.57 (0.67)	3.66 (0.54)	3.35 (0.98)	0.456			-0.07	0.890	
FU3	3.82 (0.56)	3.78 (0.49)	3.90 (0.74)	0.732			0.60	0.061	
FU4	4.00 (0.48)	3.90 (0.44)	4.27 (0.58)	0.283			1.08	0.017*	0.44
Final fusion/ maturity	3.92 (0.48)	3.89 (0.50)	4.00 (0.53)	0.753			0.76	0.184	
	Mental health								
Pre-surgery	4.27 (0.37)	4.10 (0.12)	4.60 (0.57)	0.127					
Post-surgery	4.47 (0.45)	4.37 (0.41)	4.66 (0.49)	0.180	0.30	0.300			
FU1	4.19 (1.22)	4.52 (0.39)	3.63 (1.89)	0.165			-1.12	0.085	
FU2	4.26 (0.68)	4.20 (0.71)	4.40 (0.69)	0.639			-0.25	0.521	
FU3	4.40 (0.43)	4.28 (0.44)	4.65 (0.34)	0.169			-0.08	0.711	
FU4	4.31 (0.49)	4.15 (0.42)	4.73 (0.46)	0.078			0.18	0.389	
Final fusion/ maturity	4.28 (0.54)	4.20 (0.58)	4.47 (0.50)	0.509			-0.10	0.758	
	Satisfaction								
Pre-surgery	1.33 (2.16)	0.75 (1.50)	2.50 (3.54)	0.409					
Post-surgery	3.63 (1.71)	3.42 (1.69)	4.00 (1.80)	0.488	-0.75	0.688			
FU1	3.28 (1.75)	3.00 (1.68)	3.75 (1.92)	0.426			0.32	0.732	
FU2	3.43 (1.60)	2.95 (1.67)	4.63 (0.25)	0.076			0.45	0.271	
FU3	2.96 (2.02)	2.56 (1.78)	3.75 (2.50)	0.361			-0.31	0.737	
FU4	2.55 (2.13)	2.25 (1.93)	3.33 (2.89)	0.481			-0.29	0.819	
Final fusion/ maturity	3.25 (1.84)	2.57 (1.81)	4.83 (0.29)	0.071			0.76	0.029*	0.97
	SRS-22r Total score								
Pre-surgery	4.31 (0.25)	4.29 (0.18)	4.37 (0.45)	0.758					
Post-surgery	4.33 (0.42)	4.23 (0.41)	4.50 (0.41)	0.179	0.33	0.503			
FU1	4.34 (0.31)	4.28 (0.29)	4.44 (0.33)	0.320			-0.08	0.609	
FU2	4.16 (0.50)	4.11 (0.56)	4.28 (0.34)	0.601			-0.20	0.457	
FU3	4.31 (0.35)	4.19 (0.38)	4.54 (0.08)	0.113			0.09	0.593	
FU4	4.34 (0.34)	4.22 (0.32)	4.66 (0.12)	0.053			0.28	0.180	
Final fusion/ maturity	4.30 (0.46)	4.18 (0.51)	4.57 (0.12)	0.231			0.23	0.487	

*FU* Follow-up

^*^ Statistical significance at *p* < 0.05

^ Post-hoc power analyses with α = 0.05, two-tailed, and effect size calculated for the variable at the specific time-point

For surgical outcomes evaluated by radiological parameters in Table 5, there was comparable number of cases who achieved coronal balance (83% of MCGR versus 80% of TGR, *p* = 1.000) and sagittal balance (67% of MCGR versus 75% of TGR, *p* = 0.778) at final fusion/maturity. The corrected major coronal curve after index surgery was maintained and no significant difference of changes between the two groups. Changes of T1-T12 and T1-S1 spine lengths were comparable throughout the treatment period between the MCGR and TGR groups, as well as for global kyphosis and lumbar lordosis.

**Table 5:** Inter-group comparison of radiological parameters for patients in MCGR and TGR groups

	Whole cohort (*N* = 27)	MCGR (*N* = 16)	TGR (*N* = 11)	p value	Power^	Whole cohort (*N* = 27)	MCGR (*N* = 16)	TGR (*N* = 11)	*p* value
	Good coronal balance (count in %)					Good sagittal balance (count in %)			
Pre-surgery	72%	69%	78%	1.000		62%	69%	50%	0.339
Post-index surgery	81%	85%	75%	0.618		71%	62%	88%	0.201
Final fusion	82%	83%	80%	1.000		70%	67%	75%	0.778
Parameters						Difference in changes of TGR vs MCGR (Subseq time-point—pre-surgery)_TGR_ – (Subseq time-point—pre-surgery)_MCGR_			*p* value
	Mean coronal Cobb angle of major curve, degrees (SD)								
Pre-surgery	56.3 (16.0)	51.8 (13.5)	63.5 (17.8)	0.069					
Post-index surgery	35.2 (18.0)	26.2 (6.6)	46.8 (21.6)	0.004*	0.93	-21.1 (12.4)			0.134
Final fusion	27.3 (13.0)	25.8 (11.1)	28.7 (15.1)	0.682		-28.6 (20.2)			0.379
	Mean sagittal balance, mm (SD)								
Pre-surgery	20.7 (21.4)	24.6 (22.6)	14.4 (18.9)	0.250					
Post-index surgery	28.5 (66.7)	20.5 (21.7)	41.5 (107.6)	0.497		10.3 (62.8) 7.4 (21.2)			0.246
Final fusion	16.8 (14.9)	15.0 (14.0)	19.6 (18.0)	0.661					0.630
	Mean coronal balance, mm (SD)								
Pre-surgery	13.3 (14.8)	16.4 (15.8)	8.0 (11.5)	0.177					
Post-index surgery	12.3 (12.3)	9.8 (8.8)	16.1 (16.3)	0.279		15.8 (9.7)			0.122
Final fusion	9.5 (11.8)	7.5 (12.5)	11.8 (11.8)	0.566		10.9 (9.5)			0.281
	Global kyphosis, degrees (SD)								
Pre-surgery	32.8 (20.8)	27.8 (21.2)	40.9 (18.3)	0.118					
Post-index surgery	28.0 (18.6)	23.0 (17.0)	36.2 (19.3)	0.118		-2.7 (6.9)			0.698
Final fusion	38.9 (17.7)	33.5 (18.7)	47.0 (14.6)	0.260		-15.6 (16.6)			0.374
	Lumbar lordosis, degrees (SD)								
Pre-surgery	53.5 (14.9)	54.5 (13.6)	52.0 (17.4)	0.682					
Post-index surgery	46.8 (12.3)	48.0 (15.1)	44.9 (5.9)	0.510		3.9 (8.2)			0.642
Final fusion	58.6 (12.3)	61.4 (13.7)	54.5 (10.0)	0.419		-17.1 (18.4)			0.377
	Mean T1-S1 spine length, mm (SD)								
Pre-surgery	306.3 (66.6)	332.1 (55.7)	265.1 (63.9)	0.009*	0.79				
Post-surgery	364.7 (131.3)	369.5 (44.9)	356.9 (213.7)	0.838		60.4 (123.6)			0.304
Final fusion	379.0 (63.1)	397.4 (58.7)	351.4 (67.1)	0.283		52.8 (50.5)			0.984
	Mean T1-T12 spine length, mm (SD)								
Pre-surgery	221.6 (156.2)	209.9 (34.0)	240.4 (255.4)	0.638					
Post-index surgery	218.4 (86.2)	221.7 (25.3)	213.2 (141.8)	0.833		-9.9 (196.8)			0.555
Final fusion	229.9 (44.7)	243.4 (36.7)	209.7 (53.3)	0.266		24.9 (25.8)			0.517

Subseq: Subsequent

* Statistical significance at *p* < 0.05

^ Post-hoc power analyses with α = 0.05, two-tailed, and effect size calculated for the variable at the specific time-point

Detailed examinations of HRQoL and accumulative total direct costs at specific time-points were presented in Appendix 1. Significant and moderate-to-strong correlations were found between SRS-22r total score at second follow-up visits since index surgery, however it was a negative correlation for MCGR with cost (*r*_s_ = -0.693, *p* < 0.05) but a positive correlation for TGR with cost (*r*_s_ = 0.989, *p* < 0.05). Strong negative correlations were demonstrated also for the Appearance domain scores in MCGR group, in contrast to the very strong positive correlations in patients with TGR.

## Discussion

Despite outpatient distractions, it is not uncommon for patients with MCGR to experience complications such as proximal junctional kyphosis, metallosis and distraction failures [6, 11, 12]. It is therefore important to comprehensively compare MCGR with TGR throughout the treatment period, not only in the aspects of medical costs, surgical outcomes and patient’s HRQoL, but specifically whether there is any relationship between medical expenses incurred and treatment outcomes at the corresponding time. With follow-up of this study cohort of at least 6 years until maturity, this study reveals that the greater direct medical cost of MCGR at baseline reaching cost neutrality with TGR from 2–3 years post-index surgery onwards, with comparable radiological parameters and similar SRS-22r total score changes between MCGR and TGR from preoperative to final fusion/maturity. Some important study outcome measures and their comparison reached adequate to high power, like the cost comparison, and the significantly greater increase in satisfaction of treatment (mean difference of 0.76 in domain score) by TGR patients at final fusion/maturity. The amount of intergroup differences of Appearance and Pain domain score changes (respective mean difference of 1.08 higher before fusion and 0.53 higher within first year of index surgery by TGR) also reached the minimum detectable measurement difference [27], however the statistical significance lacked power due to sample size.

Cost-effectiveness analysis is useful for assessing the gains in health relative to the costs of different health interventions [28], and for determining if the value of an intervention justifies its cost [29]. In this study, cost-effectiveness was analysed by examining the accumulative total direct medical costs of surgeries and complications throughout the treatment period, treatment outcomes based on both radiological parameters and patient’s HRQoL. This study has provided a view of costs and health benefits to reflect all stakeholders, that is, medical expenses in the healthcare system, the orthopaedic surgeons and patients. The effectiveness of the surgical interventions achieved by both TGR and MCGR is found comparable as demonstrated by the radiological evidences in this study. We meticulously evaluate the coronal and sagittal balances of the patients together with curve correction and spine length gains, and effectively assess the surgical outcomes radiologically at final fusion as compared to pre-operatively. The time-points selected are representative and that provides a more reliable examination and valid comparison of changes of spinal balances, major curve correction and increase of spine length during the lengthening period between MCGR and TGR than previous study by Akbarnia et al. [30]. Coronal balance is one of the important radiological parameters as good coronal balance can benefit patient by its positive effect on HRQoL [31].

With the comparable radiological treatment outcome, HRQoL is an outcome measure which can be optimized. Our longitudinal follow-up data suggests that at 2–3 years post-index surgery, the quality of life and accumulative direct medical cost correlated positively for TGR patients but negatively for the MCGR group. Given that the accumulative total direct medical cost of MCGR became most comparable (*p* = 0.909, and TGR: MCGR ratio = 1.010) also at exactly the time point 2–3 years post index-surgery (Fig. 1), it suggests that in spite of the increasing cost, the quality of life of TGR patients improves. On the contrary, it is an inverse relationship for the MCGR patients. These can possibly be attributed to the numerous clinical visits for rod distractions for MCGR, the discomfort patients may feel during magnetic distractions [32], and the cost of the implant when exchanges are needed. These go against the fundamental difference of the number of surgeries required between TGR and MCGR. Quality of life is often perceived as important a treatment outcome as the curve correction by the patients and caregivers. But both groups experienced similar quality of life at maturity, except the greater treatment satisfaction by TGR patients. This pattern of comparable HRQoL of TGR and MCGR patients was observed previously [33]. MCGR was not shown to have the perceivable benefits or resulting in superior HRQoL even for patients who converted from TGR to MCGR in Bauer’s study [34].

Our findings raise the questions of how we can maximize the HRQoL of patients treated with MCGR, as there is definitely room for improvement. The accumulative direct medical costs of TGR is generally higher with repeated open surgeries for distractions, but the burden of more clinic visits for magnetic rod distractions should not be underestimated. Hence we aim to make distraction visits more pleasant and convenient. Patient education and information conveyed during consultation can be considered. In addition, the accumulative costs calculated had included cost incurred for surgical complications as well as any implant failures. Although cost neutrality of MCGR to TGR was achieved by 3 years post-index surgery in this study cohort as compared to the estimated 6 years in an economic model for the United States integrated health care delivery system [35], any preventive or prophylactic measures for avoiding surgical postoperative complications for both TGR and MCGR should still be emphasized to reduce medical expenses and benefit patient care. The frequency of outpatient visits for MCGR distractions in relation to accumulated costs and any psychological burden of patients can be an area of further investigation. The regimen of monthly distractions of MCGR at our specialist clinic (with a standard distracted length of approximately 2 mm) versus those with 3-monthly or 6-monthly intervals to achieve maximum lengthening by distracting until stall or clunking [36, 37] may contribute to difference of patient’s HRQoL, and in addition how that compare to patients with TGR remains unknown.

The main limitation of this study is the difficulty in defining a cost-effectiveness ratio for the TGR and MCGR. This is because it is impractical and impossible to assign a value to the treatment outcome, whereby quantifying major curve corrected in Cobb angle is in degrees or HRQoL measure is in scores/mark per dollar. There is yet a single index measure combining the health benefits of radiological parameters and HRQoL measure or quality-adjusted life-year (QALY) [38], and the weighing of radiological measures versus the quality of life measures can vary according to surgeons or patients. Also, the 24-Item Early Onset Scoliosis Questionnaires (EOSQ-24) can be used as it is more tailored for EOS patients [39, 40]. But the generalizability of EOSQ-24 questionnaire for comparing results across countries can be limited depending on whether the questionnaire has been locally validated. One may query whether the SRS-22r questionnaire is adequate and sensitive enough to detect changes of HRQoL, however, those studies which reported similar HRQoL for both TGR and MCGR groups were using EOSQ-24 but failed to detect differences [33, 34]. The choice of a generic, utility measure such as EuroQoL 5-dimenions (EQ5D) can be coupled with disease-specific tool like SRS-22r in future studies. Moreover, the generalizability of our findings needs to be investigated as geographic locations can vary as patients and their caregivers need to travel to the clinic frequently for MCGR distractions. Future validation of findings in different countries, and multicentre studies should be useful for better understanding of the differences in overall TGR versus MCGR treatment for EOS.

## Conclusions

In conclusion, comparable treatment outcomes can be achieved by both the MCGR and TGR approach in EOS. In view of the higher accumulative total medial direct cost by the TGR and higher chance of surgical complications, future directions of clinical practice will continue to use MCGR with its benefit of distractions without surgeries as long as more than 3 years of treatment is planned. The reason for suboptimal quality of life in patients with MCGR surgery needs further study, especially in the aspect of patient’s perception of own appearance and his/her satisfaction of the intervention given.

## Data Availability

The data of this study are only available from the corresponding author upon written request and at the corresponding author’s decision.

## Appendix 1

**Table 6:** Correlations between SRS scores and total medical cost

		Whole cohort (*N* = 27)						
		Accumulative total direct medical cost						
SRS-22r		Index surgery	Index to 1-year	1-year to 2-year	2-year to 3-year	3-year to 4-year	4-year to 5-year	5-year to 6-year
Function	Pre-surgery	-0.159						
	Post-surgery	-0.438	-0.218					
	FU1	0.217	0.156	0.019				
	FU2	-0.154	-0.017	-0.355	-0.273			
	FU3		-0.383	0.031	0.385	0.387		
	FU4			0.101	0.160	0.188	0.159	0.121
	Final fusion/ maturity				0.265	0.247	0.092	0.096
Pain	Pre-surgery	-0.590						
	Post-surgery	-0.356	-0.092					
	FU1	-0.076	0.193	-0.142				
	FU2	-0.237	-0.159	-0.531	-0.300			
	FU3		-0.015	0.135	0.150	-0.039		
	FU4			0.089	0.111	0.002	-0.266	-0.188
	Final fusion/ maturity				0.035	-0.046	-0.305	-0.274
Appearance	Pre-surgery	0.640						
	Post-surgery	0.108	0.117					
	FU1	0.008	0.084	-0.172				
	FU2	0.212	0.251	-0.075	-0.202			
	FU3		-0.150	-0.168	-0.222	-0.009		
	FU4			0.089	0.181	0.196	0.095	0.020
	Final fusion/ maturity				0.212	0.117	-0.112	-0.248
Mental health	Pre-surgery	-0.635						
	Post-surgery	-0.251	0.006					
	FU1	0.380	0.329	0.237				
	FU2	-0.228	-0.122	-0.292	-0.109			
	FU3		-0.279	-0.271	0.126	0.158		
	FU4			-0.375	0.192	0.119	-0.072	-0.048
	Final fusion/ maturity				0.175	0.086	-0.081	-0.237
Satisfaction	Pre-surgery	-0.355						
	Post-surgery	-0.285	-0.162					
	FU1	-0.249	-0.373	-0.189				
	FU2	-0.568*	-0.571*	-0.274	0.167			
	FU3		-0.517	-0.194	-0.123	0.036		
	FU4			-0.280	-0.079	0.017	0.198	0.272
	Final fusion/ maturity				0.420	0.404	0.345	0.378
Total	Pre-surgery	-0.145						
	Post-surgery	-0.229	-0.025					
	FU1	-0.137	0.102	-0.252				
	FU2	-0.143	-0.027	-0.432	-0.275			
	FU3		-0.313	-0.212	0.089	0.160		
	FU4			-0.104	0.222	0.198	0.045	0.038
	Final fusion/ maturity				0.212	0.125	-0.132	-0.196
		MCGR (*N* = 16)						
		Accumulative total direct medical cost						
SRS-22r		Index surgery	Index to 1-year	1-year to 2-year	2-year to 3-year	3-year to 4-year	4-year to 5-year	5-year to 6-year
Function	Pre-surgery	-0.230						
	Post-surgery	0.097	0.172					
	FU1	0.403	0.465	-0.185				
	FU2	0.199	0.154	-0.469	-0.600			
	FU3		-0.087	0.331	0.110	0.035		
	FU4			0.170	-0.274	-0.311	-0.349	-0.401
	Final fusion/ maturity				-0.135	-0.265	-0.541	-0.512
Pain	Pre-surgery	-0.047						
	Post-surgery	0.300	0.366					
	FU1	0.412	0.497	-0.142				
	FU2	-0.086	-0.029	-0.575	-0.575			
	FU3		0.013	0.130	-0.186	-0.306		
	FU4			0.243	-0.128	-0.236	-0.527	-0.367
	Final fusion/ maturity				-0.296	-0.452	-0.799*	-0.725
Appearance	Pre-surgery	-0.875						
	Post-surgery	0.102	0.210					
	FU1	0.310	0.151	-0.337				
	FU2	0.312	0.169	-0.632*	-0.792*			
	FU3		0.209	0.026	-0.173	-0.263		
	FU4			0.184	-0.280	-0.374	-0.586	-0.747*
	Final fusion/ maturity				0.026	-0.084	-0.351	-0.580
Mental health	Pre-surgery	0.949						
	Post-surgery	0.298	0.352					
	FU1	0.140	0.273	0.526				
	FU2	-0.215	-0.192	-0.402	-0.468			
	FU3		-0.003	-0.007	0.012	-0.051		
	FU4			-0.268	-0.398	-0.448	-0.558	-0.439
	Final fusion/ maturity				0.083	0.030	-0.109	-0.330
Satisfaction	Pre-surgery	0.492						
	Post-surgery	-0.436	-0.223					
	FU1	-0.330	-0.404	-0.036				
	FU2	-0.360	-0.417	-0.119	-0.038			
	FU3		-0.697	-0.187	-0.063	-0.125		
	FU4			-0.551	-0.208	-0.164	0.036	0.184
	Final fusion/ maturity				-0.007	-0.033	-0.008	0.101
Total	Pre-surgery	-0.468						
	Post-surgery	0.311	0.357					
	FU1	0.574	0.553	-0.147				
	FU2	0.094	0.054	-0.609	-0.693*			
	FU3		0.104	0.037	-0.181	-0.293		
	FU4			0.032	-0.391	-0.462	-0.616	-0.594
	Final fusion/ maturity				-0.165	-0.300	-0.608	-0.676
		TGR (*N* = 11)						
		Accumulative total direct medical cost						
SRS-22r		Index surgery	Index to 1-year	1-year to 2-year	2-year to 3-year	3-year to 4-year	4-year to 5-year	5-year to 6-year
Function	Pre-surgery	NA						
	Post-surgery	-0.391	0.274					
	FU1	0.306	-0.106	-0.051				
	FU2	-0.607	0.888	0.963*	0.613			
	FU3		-0.034	-0.032	0.662	0.539		
	FU4			0.894	-0.199	0.549	0.921	0.925
	Final fusion/ maturity				-0.199	0.549	0.921	0.925
Pain	Pre-surgery	NA						
	Post-surgery	-0.226	0.567					
	FU1	-0.627	0.768	0.282				
	FU2	-0.681	0.380	0.112	0.704			
	FU3		0.786	0.466	0.763	0.169		
	FU4			-0.894	0.199	-0.549	-0.921	-0.925
	Final fusion/ maturity				NA	NA	NA	NA
Appearance	Pre-surgery	1.000						
	Post-surgery	0.134	-0.225					
	FU1	-0.277	0.278	0.081				
	FU2	-0.843	0.115	0.602	0.818			
	FU3		-0.848	-0.369	-0.505	0.174		
	FU4			0.060	0.749	0.998*	0.797	0.792
	Final fusion/ maturity				0.991	0.610	0.066	0.057
Mental health	Pre-surgery	NA						
	Post-surgery	-0.203	0.551					
	FU1	0.249	0.031	-0.102				
	FU2	-0.558	0.660	0.213	0.594			
	FU3		0.180	-0.488	-0.390	-0.866		
	FU4			-0.894	0.199	-0.549	-0.921	-0.925
	Final fusion/ maturity				-0.424	-0.939	-0.971	-0.969
Satisfaction	Pre-surgery	NA						
	Post-surgery	-0.160	0.555					
	FU1	0.417	-0.208	-0.252				
	FU2	0.014	0.796	0.278	0.025			
	FU3		0.034	0.032	-0.662	-0.539		
	FU4			0.835	-0.948	-0.449	0.124	0.132
	Final fusion/ maturity				0.199	-0.549	-0.921	-0.925
Total	Pre-surgery	NA						
	Post-surgery	-0.079	0.249					
	FU1	-0.439	0.451	-0.161				
	FU2	-0.983*	0.599	0.654	0.989*			
	FU3		-0.975*	-0.878	-0.596	-0.157		
	FU4			-0.835	0.948	0.449	-0.124	-0.132
	Final fusion/ maturity				0.564	-0.181	-0.698	-0.704

NA: data not available at that time point

^*^ Significant correlation with *p* < 0.05

## Abbreviations

MCGR: Magnetically controlled growing rods
TGR: Traditional growing rods
HRQoL: Health-related quality of life
EOS: Early onset scoliosis
HKD: Hong Kong Dollars
SRS-22r: Refined Scoliosis Research Society 22-item
mm: Millimetres
CI: Confidence intervals
QALY: Quality-adjusted life-year
EOSQ-24: 24-Item Early Onset Scoliosis Questionnaires
EQ5D: EuroQoL 5-dimenions
